# Perspectives and planning for retirement of Brazilian occupational physicians: an exploratory study

**DOI:** 10.47626/1679-4435-2026-1525

**Published:** 2026-02-27

**Authors:** Alberto José Niituma Ogata, Fernando Akio Mariya, Mauro Cardoso, Guilherme Augusto Murta, Junior Rezende Passos

**Affiliations:** 1 Fundação Getulio Vargas - Escola de Administração de São Paulo, Centro de Pesquisa em Administração em Saúde, São Paulo, SP, Brazil.; 2 Universidade de São Paulo, Departamento de Medicina Legal, Bioética, Medicina do Trabalho, Medicina Física e Reabilitação, São Paulo, SP, Brazil.; 3 Universidade Federal de Minas Gerais, Departamento de Medicina Preventiva e Social, Belo Horizonte, MG, Brazil.; 4 Universidade Federal do Paraná, Especialização em Medicina do Trabalho, Curitiba, PR, Brazil.; 5 Pontifícia Universidade Católica de Minas Gerais, Grupo de Pesquisa em Saúde Coletiva, Belo Horizonte, MG, Brazil.

**Keywords:** occupational medicine, retirement, occupational health, medicina do trabalho, aposentadoria, saúde ocupacional

## Abstract

**Introduction:**

Occupational medicine is a broad specialty that plays a fundamental role in protecting the workers’ health and well-being.

**Objectives:**

To analyze Brazilian occupational physicians’ perspectives on their transition to retirement, addressing aspects such as financial planning, reasons for retirement, and intended post-retirement activities.

**Methods:**

A cross-sectional descriptive study was conducted with 450 active occupational physicians in Brazil. An online questionnaire was administered to a convenience sample recruited through social media between July and August 2024.

**Results:**

Most physicians reported undertaking some form of financial planning for retirement; however, 72.00% did not consider themselves financially prepared. Furthermore, 64.40% planned to continue paid work after retirement, predominantly in consulting roles.

**Conclusions:**

These findings highlight the need for expanded financial education initiatives and structured support to support a healthy transition to retirement.

## Abstract (pt)

**Introdução:**

A medicina do trabalho é uma especialidade de amplo alcance, fundamental para a proteção da saúde e do bem-estar dos trabalhadores.

**Objetivos:**

Analisar a percepção de médicos do trabalho brasileiros acerca de sua transição para a aposentadoria, incluindo aspectos relacionados ao planejamento financeiro, aos motivos para aposentadoria e às atividades pretendidas após esse período.

**Métodos:**

Estudo descritivo transversal realizado com 450 médicos do trabalho em atividade no Brasil. Foi aplicado um questionário on-line a uma amostra de conveniência recrutada por meio de redes sociais, entre julho e agosto de 2024.

**Resultados:**

A maioria dos médicos realizava algum tipo de planejamento financeiro para a aposentadoria; entretanto, 72,00% não se consideravam financeiramente preparados. Além disso, 64,40% planejavam continuar exercendo atividades remuneradas após a aposentadoria, principalmente na área de consultoria.

**Conclusões:**

Observa-se a necessidade de ampliar iniciativas de educação financeira e de oferecer suporte estruturado para promover uma transição saudável para a aposentadoria.

## INTRODUCTION

The planning and timing of physicians’ retirement have significant implications for patients, organizations, and health care systems. Unplanned early or late retirement can have dire consequences for workforce allocation and health care service management.

Occupational medicine is a highly relevant medical specialty focused on the prevention, diagnosis, and treatment of work-related diseases and injuries. Occupational physicians play a crucial role in integrating multidisciplinary teams, contributing to workers’ health and safety ^[1]^. In Brazil, as of 2023, there were 20,804 registered occupational physicians, representing a 63.10% increase over the past decade. Of these professionals, 12,641 are over 55 years of age, with a mean age of 60 years ^[2]^. This scenario highlights the need for studies addressing the transition to retirement among these professionals.

This transition is a complex process influenced by factors such as health status, working conditions, financial planning, and personal motivations. An international study ^[3]^ indicates that poor health, low job control, and perceived organizational injustice are associated with a higher intention to retire among physicians. To our knowledge, no national studies have addressed this issue among occupational physicians.

According to a scoping review conducted in Germany, the nature and characteristics of occupational physicians’ work differ significantly from those of other medical specialties due to numerous framework conditions. Eisch et al. ^[4]^ emphasize that, in order to secure the next generation of occupational physicians, it is essential to promote occupational health through targeted support. The authors propose recommendations such as: (1) drive people- and practice-oriented research forward; (2) secure the training of the next generation through greater expansion and emphasis on occupational medicine in medical studies; (3) eliminate information deficits through continuing education in occupational medicine; (4) optimize interdisciplinary teamwork, including occupational safety specialists and family physicians; and (5) emphasize the resources of occupational physician activities to give greater priority to the issue of prevention within companies.

Furthermore, a national survey of physicians in the United States revealed that occupational physicians are predominantly engaged in clinical activities, particularly in the treatment of musculoskeletal conditions, followed by work in industry and health system management. Traditional public health approaches were less infrequent. Injured patients, employers, and healthy workers were the primary beneficiaries of these professionals’ activities. Communication about prevention and work restrictions was also commonly reported ^[5]^.

This study aims to analyze the perspective of Brazilian occupational physicians regarding their transition to retirement, addressing aspects such as perceptions and strategies related to financial planning, reasons for retirement, and planned post-retirement activities. Understanding these aspects is essential for the development of policies and strategies that support these professionals during a crucial stage of their lives. To date, we are not aware of any national publications that analyze the topic proposed in this study.

## METHODS

This is a descriptive cross-sectional study with a convenience sample of occupational physicians recruited via social media. Data were collected using an online questionnaire developed by the authors and administered by the Qualtrics platform from July 1 to August 30, 2024. The questionnaire was disseminated through social media platforms (WhatsApp, Instagram, and Facebook), using links and QR codes, and was exclusively targeted at actively practicing occupational physicians in Brazil, including with specialist certification (n = 389; 89.43%) or medical residence training (n = 65; 14.94%), with 19 respondents holding both qualifications. In 15 cases, professional credentials could not be verified; however, these participants were not excluded from the sample. The use of a convenience sample and the decision to conduct an initial univariate analysis characterize the study as exploratory.

Data collection included demographic variables, such as age, sex, place of origin, and professional training, and work-related characteristics, such as weekly working hours, type of professional activity, and participation in voluntary activities. Categorical items addressed motivation for retirement, plans for post-retirement paid activities, financial planning, and level of financial comfort. These letter variables were defined as the primary outcome (dependent) variables, while demographic and work-related variables were considered independent or explanatory variables. The study was approved by the Research Ethics Committee of Fundação Getúlio Vargas (Approval no. P.279.2024). Statistical analysis included the calculation of proportions for categorical variables, which represented most of the questionnaire items. Continuous variables, such as mean age and years of professional practice, were summarized using measures of central tendency and dispersion, including means and standard deviations. Chi-square tests were used for hypothesis testing involving categorical variables, and effect size was estimated using odds ratios. Analysis of variance was applied to continuous variables. All tests adopted a 5% significance level, consistent with standard practice in health research ^[6]^. Analyses were conducted STATA software, version 15 ^[7]^.

## RESULTS

Of the 462 individuals who accessed the questionnaire, 450 completed it in full and were included in the final analysis. Most respondents were female (52.67%) and married (69.93%). The predominant age group was 40-49 years (36.08%), followed by 30-39 years (26.50%). Nearly half of the participants (45.56%) had more than 20 years of professional experience. Most (82.22%) worked in private companies, and more than 75.00% practiced exclusively as occupational physicians.

All eligible respondents practiced occupational medicine. Of these, 388 (89.40%) had completed specialist training in occupational medicine, and 65 (14.98%) had completed medical residency in the field. A smaller proportion (n = 19; 4.38%) of professionals held both qualifications.

Most respondents (n = 370; 82.22%) worked in private companies, whereas 110 (24.40%) were employed in the public sector. Additionally, 137 (30.44%) practiced in private clinics and offices. This item allowed multiple responses. Most participants (43.75%) worked between 21 and 40 hours per week, whereas 32.81% worked more than 40 hours weekly. Overall, more than three-quarters of physicians practiced exclusively in occupational medicine.

Physicians practicing occupational medicine part-time were asked how they allocated their remaining weekly working hours. Reported activities included hospital shifts or clinical care (n = 45), private medical practice (n = 89), teaching (n = 40), lectures (n = 40), management activities (n = 37), and research (n = 7). Finally, 54 participants reported engaging in other activities unrelated to medical practice.

Table 1 presents retirement financial planning among occupational physicians. Most physicians (n = 373; 85.89%) reported undertaking some form of retirement planning, which included government pensions (Brazilian National Institute of Social Security or public service plans), private pension plans, investments, and post-retirement part-time work. Furthermore, 290 (64.44%) professionals plan to engage in paid activities following retirement.

**Table 1.** Financial planning and motivation for retirement

Retirement financial planning strategies	n	%
Government pension (INSS or public service plans)	356	79.11%
Private pension plans	260	57.78%
Financial investments	304	67.56%
Post-retirement part-time work	154	34.22%
Main factors influencing retirement decisions		
Desire for increased free-time	353	78.44%
Personal health concerns	181	40.22%
Financial issues	107	23.78%
Career dissatisfaction	76	16.89%
Work-related pressures	68	15.11%

INSS: Instituto Nacional do Seguro Social (Brazilian National Institute of Social Security).

With regard to post-retirement activities, the most frequently reported plans were to provide occupational medicine consultancy, deliver courses and lectures, engage in volunteer activities, expand leisure opportunities, and pursue hobbies and sports. Notably, more than half (n = 243; 54.00%) of physicians intend to work as consultants in occupational medicine after retirement.

Table 1 also shows the main factors influencing retirement decisions. The desire for increased free time was the most frequently cited factor (n = 353), followed by personal health concerns (n = 181). Career dissatisfaction and work-related pressures were also mentioned, although less frequently.

Nearly half of respondents (n = 228; 51.01%) reported feeling comfortable with their retirement plan. However, 72.00% (n = 324) stated that they did not feel financially prepared for retirement. Nevertheless, only 119 physicians (26.86%) sought guidance from a financial planning professional.

The main concern related to retirement was financial stability, reported by 382 respondents (84.89%), followed by health problems (n = 266; 59.10%), loss of purpose in life (n = 107; 23.78%), and social isolation (n = 73; 16.22%).

Regarding perceived quality of life, 334 (74.23%) rated it as good or very good, whereas 22 (4.89%) rated it as poor or very poor and 94 (20.89%) as fair. Likewise, 338 (75.27%) reported being satisfied or very satisfied with their practice in occupational medicine, whereas 45 (10.02%) respondents reported dissatisfaction or strong dissatisfaction and 66 (14.70%) indicated a neutral position.

Bivariate analysis was conducted to identify associations, allowing the observation of important and statistically significant relationships between response patterns.

A statistically significant association was found between perceived quality of life and retirement financial planning. Physicians who reported good or very good quality of life were more likely to have a retirement financial plan. These findings are shown in Table 2.

**Table 2.** Association between perceived quality of life and financial planning (p < 0.001)

Variable	Without financial planning	With financial planning	Total
Current quality of life			
Very good	13 (16.88%)	107 (28.69%)	120 (26.67%)
Good	28 (36.36%)	186 (49.87%)	214 (47.56%)
Fair	27 (35.06%)	67 (17.96%)	94 (20.89%)
Poor	8 (10.39%)	10 (2.68%)	18 (4.00%)
Very poor	1 (1.30%)	3 (0.80%)	4 (0.89%)
Total	77 (100.00%)	373 (100.00%)	450 (100.00%)

Career satisfaction varied according to years since graduation. The mean years (SD) since graduation was 23.32 (13.72) among very satisfied physicians and 19.73 (11.20) among satisfied physicians. In contrast, the mean years (SD) since graduation were 19.34 (1.28) among very unsatisfied physicians and 17.43 (4.76) among unsatisfied physicians (p = 0.0098). Overall, longer professional experience was associated with greater career satisfaction.

Engagement in retirement financial planning was not influenced by physician’s age. The predominant age groups were 30-39 years, representing 25.00% of those who reported financial planning and 26.90% of those who did not, and 40-49 years, accounting for 32.90% of those without planning and 36.60% of those with planning. These differences were minimal and not statistically significant (p = 0.7080).

A significant gender difference was observed in the perception of comfort regarding retirement plans. More specifically, 58.70% of men indicated comfortable with their retirement plans, compared with 44.00% of women (odds ratio 1.807; 95%CI = 1.220-2.667). Thus, men were 1.81 more likely than women to feel comfortable with retirement (p = 0.0018).

Marital status significantly influenced perceived financial preparedness for retirement (p = 0.0020). Married physicians reported higher perceived preparedness (31.85%) compared with single (14.12%) and divorced physicians (23.26%).

Physicians with longer time since graduation were more likely to plan to continue working after retirement. The mean (SD) years since graduation was 23.2 (12.8) among physicians with plans to remain professionally active, significantly higher than the mean of 16.7 (12.0) among those without such plans and 13.8 (8.44) among those who reported being unsure.

Retirement-related concerns varied according to age group. Concerns about financial stability were more frequent among younger physicians (≤ 39 years). Loss of purpose and social isolation were more frequent among older physicians, particularly those aged 60-69 years. Health concerns were observed across age groups, with no significant age-related influence. The main results are presented in Table 3.

**Table 3.** Main retirement-related concerns by age group (p < 0.001)

Age group	Financial stability	Health	Social isolation	Loss of purpose	Total
20-29	12 (3.16%)	7 (2.65%)	1 (1.39%)	4 (3.74%)	12 (2.68%)
30-39	109 (28.68%)	74 (28.03%)	11 (15.28%)	26 (24.3%)	119 (26.56%)
40-49	137 (36.05%)	89 (33.71%)	20 (27.78%)	29 (27.1%)	161 (35.94%)
50-59	63 (16.58%)	52 (19.70%)	20 (27.78%)	21 (19.63%)	81 (18.08%)
60-69	45 (11.84%)	33 (12.50%)	18 (25.00%)	24 (22.43%)	59 (13.17%)
≥ 70	14 (3.68%)	9 (3.41%)	2 (2.78%)	3 (2.8%)	16 (3.57%)
Total	380 (100.00%)	264 (100.00%)	72 (100.00%)	107 (100.00%)	448 (100.00%)
	p = 0.022	p = 0.753	p = 0.002	p = 0.021	

No significant association was observed between perceived quality of life and participation in volunteer activities (p = 0.1420). Among those who rated their quality of life as good or very good, 28.70% reported participation in volunteer activities, a proportion statistically similar to the 31.60% observed among those who rated their quality of life as fair to very poor.

## DISCUSSION

The present study showed that, although most occupational physicians reported undertaking some form of financial planning for retirement, a significant portion did not feel financially prepared, even while reporting reasons to leave professional practice, such as increased leisure time, health considerations, job dissatisfaction, and daily work pressures. Participants reported post-retirement planning strategies that included reliance on the government pension system, as well as financial investments, private pension plans, and the possibility of continuing professional activity after retirement.

An international study involving five countries found that, after leaving full-time employment, 85.00% planned to work part-time, 48.00% aimed to engage in consulting activities, and 34.00% intended to hold part-time leadership positions. Although 60.00% reported having financial planning for retirement, 12.00% had not yet begun to consider the issue. Only 35.00% indicated being comfortable with their retirement plans ^[8]^.

Conversely, a study of retired physicians showed that they generally exhibited high levels of life satisfaction, which increased proportionally with time since retirement ^[9]^. According to these physicians, the loss of their professional role represented the main challenge during the transition to retirement.

A mixed-method study of physicians working in academic settings identified that key barriers to retirement planning included inadequate personal financial management, inflexible institutional structures, and professional norms. Identified facilitators comprised financial planning resources tailored to physicians at different career stages, opportunities and support for late-career transitions, mentorship programs in the later stage of practice, support for intergenerational collaboration, and institutional recognition of retired physicians ^[9]^.

However, early retirement may be associated with low job satisfaction, medicolegal issues, health concerns, and financial troubles. Low job satisfaction may involve perceptions of low job control, low morale, and dissatisfaction with the internal justice system of medicine as a self-regulated profession. Such disillusionment was expressed by a sense of frustration with colleagues, feeling undervalued, lacking prestige, and loss of interest in their work. Furthermore, experiencing poor health, cognitive decline, difficulty sleeping, and psychological distress were also factors leading to an early physician’s retirement ^[10]^.

The predominance of physicians who plan to continue engaging in paid work after retirement aligns with global trends toward extended working lives, particularly among highly qualified professionals. Occupational medicine consultancy was the most frequently cited activity, suggesting that physicians value maintaining professional ties to their field even after retirement. Similar patterns have been observed in other countries, where health care professionals seek to remain active in order to preserve their sense of purpose and social contribution ^[11]^.

Concerns about financial stability and personal health underscore the need for policies that support occupational physicians during transition to retirement. Only one quarter of physicians sought assistance from a financial planning specialist. Financial education programs, psychological support, and promotion of a healthy lifestyle may contribute to a more secure and satisfying retirement. Furthermore, the World Health Organization ^[12]^ emphasizes the importance of healthy aging and the maintenance of functional capacity.

According to Silver et al., understanding when physicians plan to retire and how they intend to transition out of clinical practice has been shown to facilitate succession planning. Institutions may consider implementing retirement mentorship programs, developing structured resource toolkits, offering educational sessions, and financial planning guidance throughout physicians’ careers, and creating post-retirement opportunities that preserve institutional ties through teaching, mentorship, and peer support ^[10]^.

This study employed a convenience sample that does not compromise the analyses but limits representativeness of the population of Brazilian occupational physicians.

## CONCLUSIONS

The present study demonstrates that the transition to retirement among Brazilian occupational physicians is influenced by factors such as financial planning, personal health status, and professional motivations. Although most physicians undertake some form of retirement planning, many do not feel financially prepared. The continuation of paid work after retirement, particularly in consulting roles, is a common trend. It can be concluded that financial education, integrated with retirement planning, should begin early in physicians’ career in order to promote realistic planning aligned with personal goals, rather than being postponed until the years immediately preceding retirement. The implementation of policies and programs to support physicians during the transition to retirement is recommended, including financial education, psychological support, and promotion of healthy habits.
